# Urinary amylase / urinary creatinine ratio (uAm/uCr) - a less-invasive parameter for management of hyperamylasemia

**DOI:** 10.1186/1471-2431-13-205

**Published:** 2013-12-13

**Authors:** Keita Terui, Tomoro Hishiki, Takeshi Saito, Tetsuya Mitsunaga, Mitsuyuki Nakata, Hideo Yoshida

**Affiliations:** 1 Department of Pediatric Surgery, Graduate School of Medicine, Chiba University, 1-8-1 Inohana, Chuo-ku, Chiba, Japan

**Keywords:** Hyperamylasemia, Pancreatitis, Choledochal cyst, Amylase, Creatinine, Urine

## Abstract

**Background:**

The serum level of amylase (sAm) is commonly used as a biochemical marker for diagnosis and management of pancreatic disorders. However, the use of the urine level of amylase (uAm) is limited in practice, because the diagnostic ability of uAm is inferior to that of sAm. In the present study, the possible concordance of uAm-rerated parameters with sAm was investigated, and evaluate the usefulness of uAm for management of hyperamylasemia.

**Methods:**

From June 1995 to October 2009, 804 samples of both urine and blood were collected from 128 patients in order to measure the serum level of amylase (sAm) and the urine level of amylase (uAm) and creatinine (uCr). Concordance of parameters using uAm compared to sAm was assessed. Parameters used were uAm, amylase creatinine clearance ratio (ACCR), and the ratio of uAm to uCr (uAm/uCr).

**Results:**

uAm/uCr had the best correlation with sAm (r = 0.779, p < 0.001) compared to uAm (r = 0.620, p < 0.001) and to ACCR (r = 0.374, p < 0.001), when sAm was over the standard level. The area under the receiver operating characteristic curve of uAm/uCr (0.884) was significantly higher than that of uAm (0.766) and of ACCR (0.666) (p < 0.001 for each). The cutoff value of uAm/uCr was 569.8, with a sensitivity of 81.0% and a specificity of 83.1%.

**Conclusions:**

The uAm/uCr ratio correlated with sAm, and may be an alternative to sAm for prediction of hyperamylasemia. Use of urine samples results in a decreased need for blood sampling, which is especially beneficial in pediatric patients.

## Background

The serum level of amylase (sAm) is commonly used as a biochemical marker for diagnosis and management of pancreatic disorders [1-3]. Amylase is also one of only a few serum enzymes that are detectable in urine due to its small size and glomerular filtration [4]. However, the use of the urine level of amylase (uAm) is limited in practice [5], because the diagnostic ability of uAm is inferior to that of sAm [6,7]. In a few reports, uAm has been used as a marker after endoscopic retrograde cholangiopancreatography or pancreas transplantation [8,9]. The amylase creatinine clearance ratio (ACCR) is an index that uses uAm. ACCR is known to increase during pancreatitis; however, it has little diagnostic value because of its low specificity and sensitivity [5,7].

Since 1990s, we have pursued possibility of using urine samples for management of pancreatitis, in order to decrease occasions of blood sampling. Index using uAm, however, had little scientific basis. In the present study, the possible concordance of uAm with sAm was investigated. If a urine sample is equally or more useful and reliable to measure a biochemical parameter, then patients can be subject to fewer blood draws in the management of hyperamylasemia. This is especially relevant in pediatric patients. For parameters possibly correlated with sAm, the ratios ACCR and uAm/uCr (the corrected value of uAm divided by the urine level of creatinine (uCr)) were adopted in the present study. The concept of uAm/uCr is based on other methods that are used to evaluate excretion of various enzymes into the urine [10,11].

## Methods

**Figure 1:**
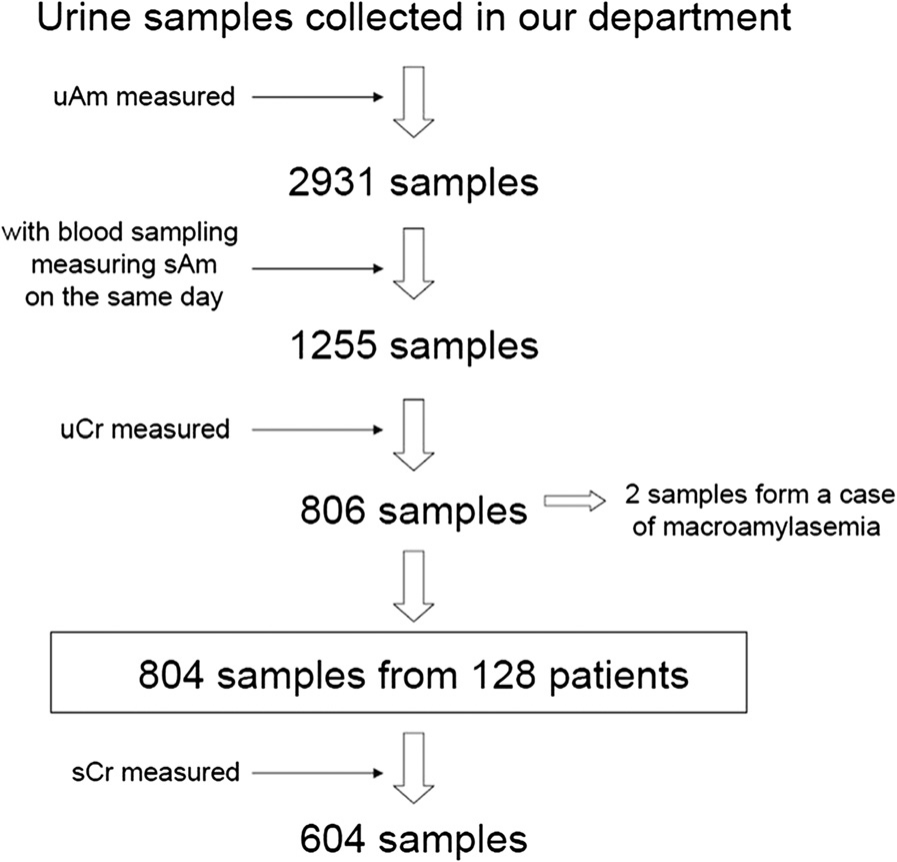
A flow chart of data collection.

Retrospective, observational study was conducted in the single tertiary referral center, from June 1995 to October 2009. A flow chart of data collection is shown in Figure 1. Firstly, 2931 urine samples of uAm obtained in our department were extracted from hospital databases. In these, 1255 samples were obtained with blood sampling measuring sAm on the same day of urine sampling. And in these, 806 samples were also taken with uCr simultaneously. One case of macroamylasemia (with 2 samples) was excluded. Diseases in the salivary glands were not included in the present study. Eventually, total of 804 urine and blood samples which contained uAm, sAm and uCr were collected from 128 patients.

Urine samples were collected with the spot collection technique. The timings of sample collection were various and also independent from those of blood sampling. In neonates and infants, urine was sampled by using disposable Pediatric Urine Collector (Atom®).

Both sAm and uAm were determined by an enzymatic method using blocked p-nitrophenyl-α-maltoheptaoside as a substrate. uCr was determined by an enzymatic method involving creatinase, sarcosineoxidase and peroxidase. Standard levels of sAm and uAm in our institution are set at 35–110 IU/L and <450 IU/L, respectively.

ACCR was calculated in 604 samples (75% of all samples), because not all blood samples were tested for the serum level of creatinine (sCr). ACCR was determined independently of urine volume and time of sampling by using the formula (uAm / sAm) / (uCr / sCr) [12]; uAm/uCr was calculated simply by dividing uAm by uCr.

Statistical analyses were performed by using software (statistical computing; R Foundation for Statistical Computing, Vienna, Austria). Differences between groups were tested by means of the Student’s t-test or the Mann–Whitney U test. Associations were assessed by Pearson’s correlation coefficient test or Spearman’s rank correlation and expressed as the corresponding correlation coefficient, r. Simple regression analysis was used to evaluate correlations between sAm and parameters of urinary amylase: uAm, ACCR, and uAm/uCr. The abilities of the three parameters of urinary amylase to predict abnormalities of sAm were assessed with receiver operating characteristic (ROC) analysis. The area under the curve of each ROC was calculated, and the difference between each pair was assessed using the critical ratio. The cutoff value was derived by the point with shortest distance to the point with a true positive fraction of 100% and a false positive fraction of 0% on the ROC curve. All results are expressed as mean ± standard deviation. P values of <0.05 were considered statistically significant.

Informed consent for participation in the study was not obtained because all data were collected as part of routine diagnosis and treatment, and analyzed retrospectively. The study protocol was approved by the institutional ethics committee of Chiba University.

## Results

The median age of patients at the sampling was 8.1 years old (range, 2 months-29 years). The level of amylase in each age-group is shown in Table 1. Patients were divided into 4 groups: babies (<1 year old, n = 18), infants (1–5 years old, n = 266), schoolchildren (6–12 years old, n = 330) and adolescents (>13 years old, n = 190 including 22 twenties). Normal sAm was present in 94.4% of babies, 60.5% of infants, 60.3% of schoolchildren, and 76.3% of adolescents.

**Table 1:** The level of amylase in age-groups

	**Age (year)**	**No.**	**Serum amylase (% of normal level)**		**Urine amylase**
Babies	< 1	18	33.5 ± 33.6	(94.4%)	56.6 ± 47.0
Infants	1 – 5	266	178.1 ± 248.9	(60.5%)	600.6 ± 938.7
Schoolchildren	6 – 12	330	183.2 ± 269.5	(60.3%)	755.8 ± 2253.9
Adolescents	> 13	190	105.6 ± 91.2	(76.3%)	438.8 ± 616.7
					(IU/L)

Amylase concentrations in different clinical conditions are shown in Table 2. Choledochal cyst was the diagnosis with the largest number of patients in the present study with 529 samples (65.8% of all samples). In patients with choledochal cyst, hyperamylasemia can often be induced by a protein plug stuck in the common channel [13]. The samples were collected during not only the perioperative period, but also during outpatient follow-up; therefore, choledochal cyst samples included a large number of normal sAm values. Normal sAm accounted for 75.6% of the choledochal cyst and 70.7% of the non-pancreatic disease samples. Tumors in the present study included pancreatic tumors (solid pseudopapillary tumors of the pancreas, n = 2; pancreatoblastoma, n = 1), and non-pancreatic tumors in the upper abdominal area (neuroblastoma, n = 5; lymphatic tumor, n = 2; Wilms’ tumor, n = 1; germ cell tumor, n = 1). Pancreatic duct obstruction consisted of pancreatolithiasis (n = 2) and sphincter of Oddi dysfunction (n = 1). Samples from patients with “non-pancreatic disease” were those that were collected for screening of abdominal pain which turned out to be of non-pancreatic origin. Non-pancreatic disease includes abdominal pain of unknown origin (n = 14), ulcerative colitis (n = 3), gallbladder stone (n = 2), urinary tract infection (n = 1) and SMA syndrome (n = 1).

**Table 2:** The level of amylase in different clinical conditions

	**No.**	**Serum amylase (% of normal level)**		**Urine amylase**
Choledochal cyst	529	125.0 ± 185.5	(75.6%)	485.8 ± 1547.4
Tumor	87	196.1 ± 216.9	(44.8%)	598.0 ± 1084.5
Trauma	79	370.9 ± 429.7	(39.2%)	1435.8 ± 2195.4
Pancreatic duct obstruction	47	178.7 ± 177.8	(57.4%)	686.5 ± 1824.4
Pancreatitis	21	138.2 ± 62.4	(19.0%)	725.6 ± 845.5
Non-pancreatic disease	41	115.7 ± 110.7	(70.7%)	576.1 ± 766.0
				(IU/L)

### Association between sAm and urinary amylase parameters

**Figure 2:**
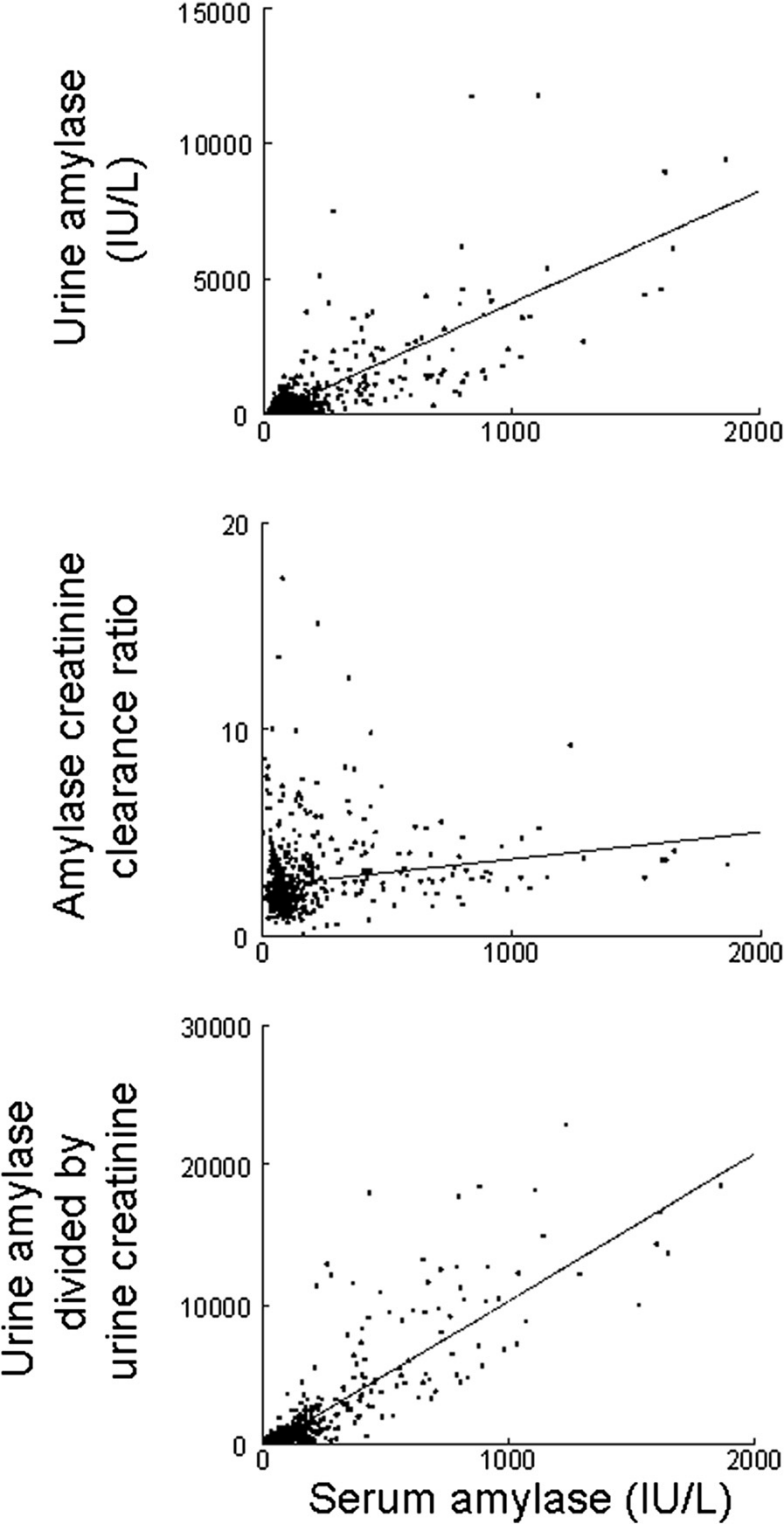
**Association between the serum level of amylase and the urine level of creatinine, amylase creatinine clearance ratio, and the level of urinary amylase divided by urine creatinine.** The regression line is drawn in each.

Association of sAm with uAm, ACCR, and uAm/uCr are shown in Figure 2A, 2B, and 2C, respectively. The regression line is drawn in each figure. The regression lines of uAm, ACCR and uAm/uCr are represented by the equations sAm = (0.090 × uAm) + 104.8 (r = 0.610, p < 0.001), sAm = (22.1 × ACCR) + 116.7 (r = 0.168, p < 0.001), and sAm = (0.070 × uAm/uCr) + 62.9 (r = 0.859, p < 0.001), respectively.

With regards to correlation analysis, uAm/uCr had the best correlation with sAm (r = 0.667, p < 0.001) compared to uAm (r = 0.508, p < 0.001) and ACCR (r = 0.167, p < 0.001). In 273 samples (34.0% of all samples), sAm was over the standard level (>110 IU/L). When sAm was over the standard level, uAm/uCr also had the best correlation with sAm (r = 0.779, p < 0.001) compared to uAm (r = 0.620, p < 0.001) and ACCR (r = 0.374, p < 0.001). When sAm was normal, all the three parameters did not correlate well with sAm (uAm, r = 0.245, p < 0.001; ACCR, r = −0.218, p < 0.001; uAm/uCr, r = 0.289, p < 0.001).

In 11 samples (1.4% of all the samples), sCr was over the standard level (>1.0 mg/ml). When sCr was over the standard level, uAm/uCr was 1402.0 ± 2854.1 and correlated with sAm (r = 0.699, p = 0.025). When sCr was normal, uAm/uCr was 181.0 ± 50.3 and also correlated with sAm (r = 0.665, p < 10^-12^).

Correlations between sAm and uAm/uCr were assessed by different age groups and diseases (Table 3). In infants and schoolchildren, all the factors had correlations between sAm and uAm/uCr, while none had correlation in babies. In adolescents, correlations were observed in pancreatic duct obstruction and non-pancreatic disease.

**Table 3:** Correlation between sAmy and uA/uC by different age-groups and diseases

	**Babies**	**Infants**	**School children**	**Adolescents**	**Total**
Choledochal cyst	0.331	0.743^**^	0.636^**^	0.538^**^	0.605^**^
Tumor	-	0.829^**^	0.859^**^	0.522^**^	0.715^**^
Trauma	-	0.939^**^	0.925^**^	na	0.890^**^
Pancreatic duct obstruction	-	-	0.923^**^	0.725^**^	0.794^**^
Pancreatitis	na	-	na	0.582^**^	0.586^**^
Non-pancreatic disease	na	0.713^**^	0.784^**^	0.901^**^	0.827^**^
Total	0.588^*^	0.769^**^	0.764^**^	0.560^**^	0.779^**^

The each value represents the corresponding coefficient of correlation (r).

*:p<0.05, **:p<0.01.

na; not assessed due to small samples (n<10).

### Prediction of sAm abnormalities with urinary amylase parameters

**Figure 3:**
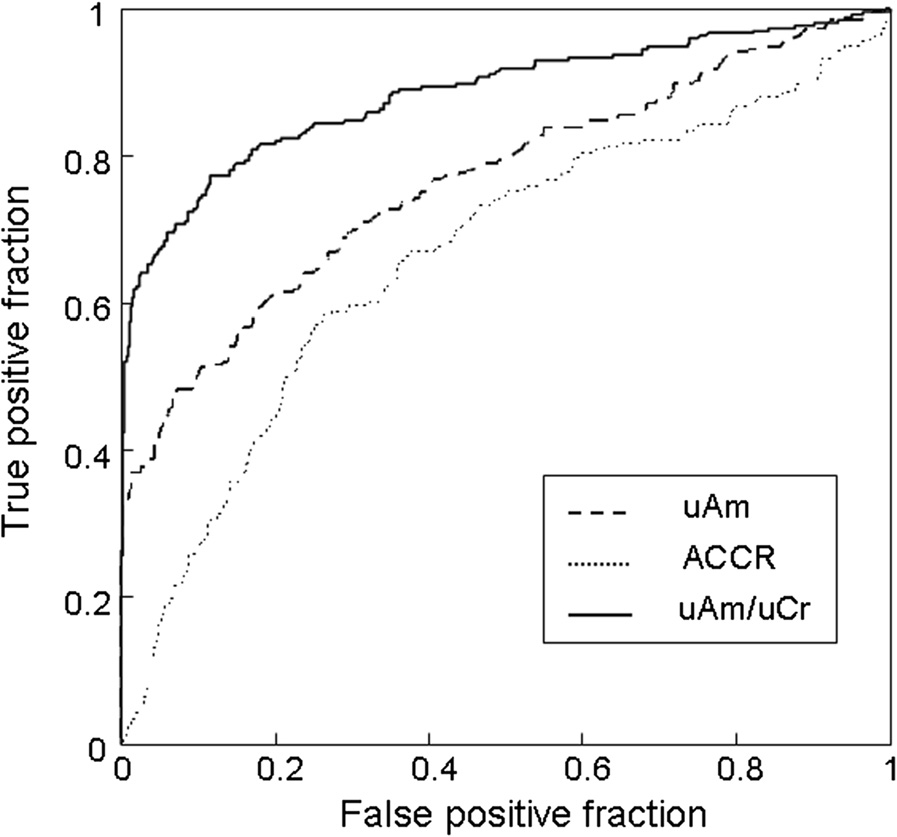
**Receiver operating characteristic curves for the urine level of creatinine (uAm, broken line), amylase creatinine clearance ratio (ACCR, dotted line) and the level of urinary amylase divided by urine creatinine (uAm/uCr, solid line) as predictors of abnormal level of serum amylase.** The area under the curve for uAm/uCr is significantly greater than that for uAm and ACCR.

ROC curves are plotted for abnormal sAm (>110 IU/L) in Figure 3. The area under the ROC curve of uAm/uCr (0.884) was significantly higher than that of uAm (0.766) and ACCR (0.666) (p < 0.001 for each). The cutoff value of uAm/uCr was 569.8 with a sensitivity of 81.0% and a specificity of 83.1%.

## Discussion

In the diagnosis and management of pancreatic disorders, measurement of sAm has played a central role since Elman et al. demonstrated the value of sAm in diagnosis of pancreatitis [5,14]. However, it is also true that sAm has problems with respect to diagnostic ability. Estimates of the sensitivity of amylase range from 60% to 98% [15,16], although it is difficult to estimate because elevated sAm itself is frequently used as a diagnostic criterion [5,17,18]. Furthermore, almost 40% of patients under 3 years of age with a diagnosis of acute pancreatitis were reported to have normal amylase but elevated lipase levels [19]. This erratic low sensitivity of sAm has been explained as resulting from the short half-life of amylase in the serum [1-3,15]. The specificity of sAm is also not high enough to use as a screening parameter for pancreatic disorders. While hyperamylasemia is often seen in various pancreatic diseases, extrapancreatic factors also result in hyperamylasemia (salivary disease, gastrointestinal disease, gynecologic disease, extra-pancreatic tumors, and others) [1-3]. Hyperamylasemia was found in 9.7% of patients with appendicitis [20], 9.3% of patients with acute cholecystitis [21], and 16.0% of patients with a perforated peptic ulcer [22]. To compensate for the incomplete diagnostic ability of sAm, other pancreatic enzymes have been proposed. In particular, the serum level of lipase is a comparable marker of pancreatic disease, but rapid and cost-effective measurement systems are not broadly available [1-3]. Urinary trypsinogen-2 strip test has also drawn attention recently for early detection of pancreatitis, but is not quantitative and less sensitive [23,24]. Therefore, sAm has some issues concerning its use, but has been used broadly as practical marker of pancreatic disorders.

Amylase has a relatively small molecular weight of about 50,000 Daltons; therefore, it passes through the glomerulus and is one of only a few serum enzymes detectable in urine [1]. Therefore, uAm can potentially be used as a marker of hyperamylasemia. The purpose of the present study was to assess the usefulness of urine samples in this regard. The leading reason for conducting this research was that if urine sample is more useful and reliable, then patients can undergo a urine test as an alternative to a blood test. Collection of urine is less invasive and generally leads to less suffering than blood sampling, which can be especially stressful in pediatric patients.

The parameters investigated from urine samples in the present study were uAm, ACCR and uAm/uCr. First of all, uAm can be easily obtained from urine samples. By using a 1-hour value, a superior sensitivity of uAm was reported in 1963 [25]. However, uAm has recently been reported to have no further advantages in the diagnosis of pancreatitis compared to sAm [7,16]. Even if amylase activity per hour is used as normalization, the diagnostic performance of uAm is worse than that of sAm [6]. Second, ACCR was developed to differentiate pancreatitis from hyperamylasemia caused by other conditions. The concept was based on the fact that amylase clearance increases significantly in acute pancreatitis, while creatinine clearance does not [26-28]. However, ACCR also has problems with specificity and sensitivity, and is considered to be less important than sAm [7,15,28-30]. Third, uAm/uCr is the corrected value of uAm divided by uCr. The concept of uAm/uCr is based on the evidence that the variabilities of enzyme excretion into the urine are the smallest when the results are expressed as the ratio of enzyme activity / uCr [31,32]. Amylase passes through the renal filtration circuit primarily without passive elimination or reabsorption [33,34]. Therefore, uAm satisfies the criteria for the use of the correction by uCr [11]. Another merit of uAm/uCr is that it can be obtained from a urine sample, and a blood sample is not required.

In the present study, among the three parameters of urinary amylase (uAm, ACCR, and uAm/uCr), uAm/uCr had the best correlation with sAm especially in the patients with hyperamylasemia, and the best ability to predict an abnormal elevation of sAm. This result suggests the potential use of uAm/uCr as an alternative for sAm. Furthermore, details of the correlation between sAm and uAm/uCr were evaluated in order to elucidate the characteristics of uAm/uCr. The correlation between sAm and uAm/uCr was low in babies, and was significant in infants and schoolchildren. This indicates that the level of amylase itself cannot be used in babies, as discussed later. The low correlation in adolescents can be explained by the high rate of normal sAm in adolescent samples in the present study. In the range of normal sAm, correlation between sAm and uAm/uCr tended not to be significant. A number of normal samples were also collected during follow-up for a choledochal cyst, and also resulted in a low correlation. Low correlation in pancreatitis probably results from a low mean sAm (115.7 IU/L) despite the low rate of normal sAm in this population. Except for these conditions, correlations were relatively high in a number of various other conditions, including tumor, trauma, pancreatic duct obstruction, and non-pancreatic disease. Also, in renal failure with elevated sCr, the correlation between sAm and uAm/uCr was significant. Therefore, uAm/uCr could be applicable for various conditions of hyperamylasemia after the first year of life, and does not appear to be influenced by elevated sCr. In the management of hyperamylasemia, uAm/uCr can potentially be used not for diagnosis but as a marker for following up on levels of amylase.

In the pediatric population, the level of amylase activity increases with age [1,15]. Amylolytic enzyme activity was found to be feeble in children under 1 year of age, and exceeded the average range for adults beyond the first year [35]. The adult level of pancreatic amylase activity in duodenal juice is reached at the age of 18 months [36]. In serum, the amylase level reaches the normal adult level by the age of 8 months [37]; however, the major component of amylase originates from the salivary glands. Pancreatic amylase activity in serum reaches the normal adult level by 7 to 10 years of age [38,39]. In contrast, the pancreatic proportion of urinary amylase approaches the adult level by 16 months of age [40]. These age-dependent specificities should be considered when interpreting data surrounding sAm and uAm/uCr in a pediatric population.

A limitation of this study is the characteristics of study population; because the subjects were patients from our department of pediatric surgery, the study population does not match common etiologies of pediatric hyperamylasemia in the greater population [19]. In the present study, the number of the patients with choledochal cyst was quite high, and only a few cases of pancreatitis from systemic causes were investigated. Thus, further investigations are needed in different study populations. The method of urine sampling is also a limitation. The recommended urine sampling technique is the second morning urine after voiding the night urine, in order to avoid the influences of diurnal rhythms of enzyme excretion and collection errors [31]. In the present study, however, the time of day of urine sampling was not constant, and was also not constant in relation to the time of blood sampling. These issues of sample collection caused unavoidable measurement error, but they were considered acceptable by limiting the time of urine and blood sampling to the same day and by including a large number of samples. Furthermore, uAm tends to peak later and maintain high level longer than sAm [15,41]. These physiological differences also should be considered in interpreting the data.

## Conclusions

uAm/uCr was found to significantly correlate with sAm, and therefore may be of use as an alternative to sAm during management of patients with hyperamylasemia. Use of urine samples results in a decreased number of blood samples, which is especially important in pediatric patients. This retrospective preliminary report will need to be supported by further studies in order to elucidate the practical usefulness of uAm/uCr in management of hyperamylasemia.

## Abbreviations

sAm: The serum level of amylase; uAm: The urine level of amylase; uCr: The urine level of creatinine; sCr: The serum level of creatinine; ACCR: The amylase creatinine clearance ratio; uAm/uCr: The corrected value of uAm divided by uCre; ROC: Receiver operating characteristic.

## Competing interests

The authors declare that they have no competing interests.

## Authors’ contributions

KT, TH, TS, TM and MN collected samples; KT performed the statistical analysis and wrote the manuscript; and HY designed the study and reviewed the manuscript. All the authors have read and approved the final manuscript.

## Pre-publication history

The pre-publication history for this paper can be accessed here:

http://www.biomedcentral.com/1471-2431/13/205/prepub
